# Protecting a transgene expression from the HAC-based vector by different chromatin insulators

**DOI:** 10.1007/s00018-013-1362-9

**Published:** 2013-05-16

**Authors:** Nicholas CO Lee, Artem V. Kononenko, Hee-Sheung Lee, Elena N. Tolkunova, Mikhail A. Liskovykh, Hiroshi Masumoto, William C. Earnshaw, Alexey N. Tomilin, Vladimir Larionov, Natalay Kouprina

**Affiliations:** 1 Laboratories of Molecular Pharmacology, National Cancer Institute, Bethesda, MD 20892 USA; 2 Institute of Cytology, Russian Academy of Sciences, St. Petersburg, Russia; 3 Laboratory of Cell Engineering, Department of Human Genome Research, Kazusa DNA Research Institute, 2-6-7 Kazusa-Kamatari, Kisarazu, Chiba 292-0818 Japan; 4 Wellcome Trust Centre for Cell Biology, University of Edinburgh, Edinburgh, EH9 3JR Scotland, UK

**Keywords:** Insulator, tDNA-gamma-satellite, cHS4, Human artificial chromosome-HAC

## Abstract

Human artificial chromosomes (HACs) are vectors that offer advantages of capacity and stability for gene delivery and expression. Several studies have even demonstrated their use for gene complementation in gene-deficient recipient cell lines and animal transgenesis. Recently, we constructed an advance HAC-based vector, alphoid^tetO^-HAC, with a conditional centromere. In this HAC, a gene-loading site was inserted into a centrochromatin domain critical for kinetochore assembly and maintenance. While by definition this domain is permissive for transcription, there have been no long-term studies on transgene expression within centrochromatin. In this study, we compared the effects of three chromatin insulators, cHS4, gamma-satellite DNA, and tDNA, on the expression of an *EGFP* transgene inserted into the alphoid^tetO^-HAC vector. Insulator function was essential for stable expression of the transgene in centrochromatin. In two analyzed host cell lines, a tDNA insulator composed of two functional copies of *tRNA* genes showed the highest barrier activity. We infer that proximity to centrochromatin does not protect genes lacking chromatin insulators from epigenetic silencing. Barrier elements that prevent gene silencing in centrochromatin would thus help to optimize transgenesis using HAC vectors.

**Electronic supplementary material:**

The online version of this article (doi:10.1007/s00018-013-1362-9) contains supplementary material, which is available to authorized users.

## Introduction

Gene therapy requires the provision of a wild-type copy of a gene to a gene-deficient cell in order to restore the proper expression and function of the damaged gene. Key requirements for successful gene therapy include: (1) the use of a full-length gene (or groups of genes) with all its regulatory elements, (2) a safe and effective vector to deliver the therapeutic gene to specific patient cells, and (3) stable long-term gene expression in a physiologically regulated fashion.

At present, full-length genes are available in characterized clones from human BAC and YAC libraries, or alternatively a desired gene can be selectively isolated from human genomic DNA using the transformation-associated recombination (TAR) cloning procedure, which exploits the high level of homologous recombination in yeast cells [1–3]. *H*uman *a*rtificial *c*hromosomes or HAC-based vectors are gene-delivery vectors with promising advantages over currently used viral vectors [4–12]. All HACs by definition contain a functional centromere and can therefore replicate and segregate like normal chromosomes in human cells, without the need for integration into the host genome. Also, HAC vectors have essentially unlimited cloning capacity and are therefore able to carry genes or genomic fragments up to several Mb in extent. Finally, because they do not encode foreign proteins, HAC vectors minimize adverse host immunogenic responses and the risk of cellular transformation. Several studies have demonstrated the use of HAC-based vectors for delivery and expression of genomic copies of genes in patient-derived gene-deficient cell lines and in mice ([10, 13–16] and references therein). The competence of HACs for transgenesis is favored by the presence of a unique recognition site for site-specific recombinases such as Cre, FLP, and phiC31 [17]. The first HAC vectors containing site-specific recombination sites for gene loading have been constructed using 21HAC engineered from the human chromosome 21 [10, 18].

One of the most versatile current HAC-based vectors is an alphoid^tetO^-HAC that was engineered from a synthetic alphoid DNA array in human fibrosarcoma HT1080 cells [19]. This HAC comprises a circular megabase-size alphoid DNA array in which a single copy gene acceptor site was inserted [19–21]. The gene-loading site is within a centrochromatin domain containing CENP-A nucleosomes interspersed with nucleosomes rich in histone H3 dimethylated at lysine 4 [22] and thus permissive for transcription [23]. Uniquely, this HAC can be easily eliminated from cell populations by inactivation of its conditional kinetochore [20, 24–27, 29]. HAC elimination may therefore be used to control for phenotypic changes attributed to expression of HAC-encoded genes [22]. The HAC remains structurally stable during months of propagation in host cells and does not integrate into the host chromosomes [21, 22]. Although the alphoid^tetO^-HAC thus fulfills criteria for an appropriate gene-delivery vector, a detailed analysis of long-term gene expression from this HAC has been lacking.

**Fig. 1:**
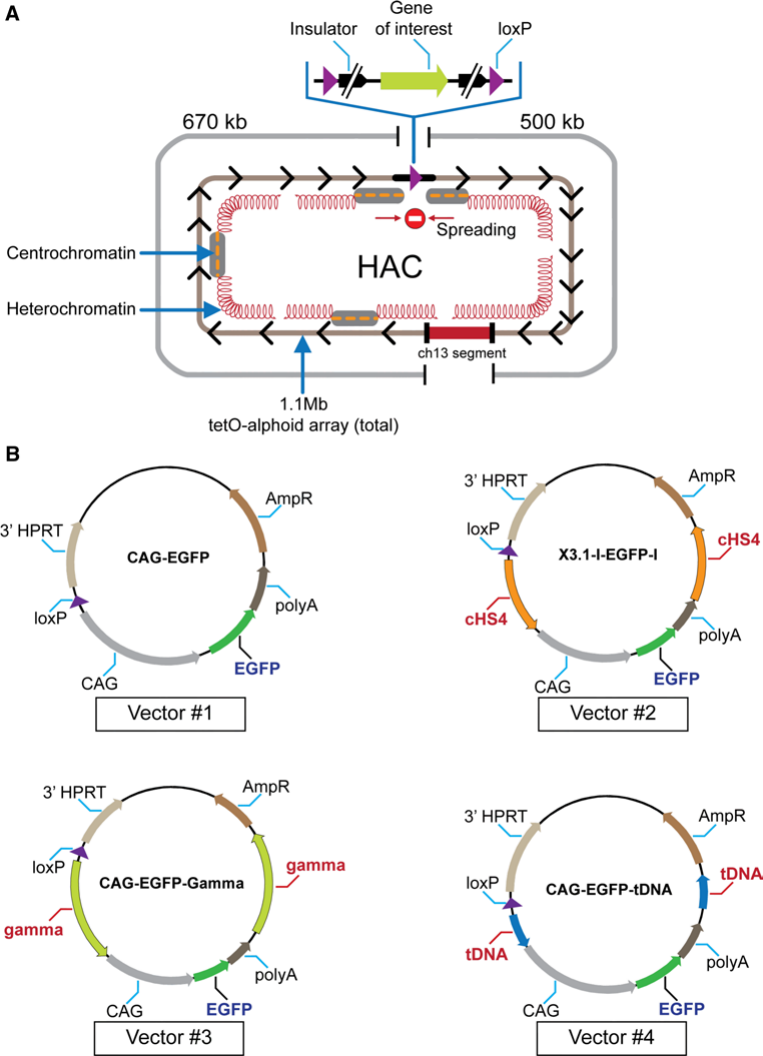
**a** Diagram of the alphoid^tetO^-HAC with a unique gene loading loxP site [20]. The HAC contains <6,000 copies of the 42-bp tetracycline operator (tetO) sequence incorporated into every second alphoid DNA monomer of the 1.1 mega-base size alphoid DNA array [19, 21]. Because tetO sequences are bound with very high affinity and specificity by the tet repressor (tetR), the tetO sequences in the HAC can be targeted efficiently in vivo with tetR chromatin modifier fusion proteins. This can result in HAC elimination from cell populations by inactivation of its conditional kinetochore [19]. A gene-loading site is inserted into a transcriptionally permissive centrochromatin domain that is flanked by heterochromatin domains essential for centromere function. A repositioning of a heterochromatin domain(s) may potentially lead to silencing of genes loaded into the HAC. **b** Diagrams of the vectors used in this study. All vectors contain an *EGFP* color marker under a control of *CAG* promoter and a 3′ HPRT-loxP module. In three vectors (#2, #3, and #4), *EGFP* is flanked either by cHS4, gamma-satellite DNA or tDNA sequences, correspondingly. Vector #1 has no insulator sequence. The vectors were inserted into the loxP site of the alphoid^tetO^-HAC propagated in HPRT-deficient hamster CHO cells

For stable physiological expression of a gene of interest in centrochromatin, the expressed gene should not disturb the centrochromatin itself (because its disruption may induce inactivation of the HAC kinetochore followed by HAC loss) [19, 24]. Also, the inserted gene should evade silencing potentially induced as heterochromatin domains flanking centrochromatin in all centromeric regions spread across the gene acceptor site [27, 28] (Fig. 1a). A logical strategy to overcome problems caused by shifting chromatin dynamics in the HAC centromere is to flank the transgene with insulator sequences as is routinely done for genes expressed from viral vectors [29–31].

In this study, to clarify a role of insulators in protecting gene expression from the HACs, we compared an effect of each of several candidate insulator sequences—cHS4, gamma-satellite DNA, and a cluster of *tRNA* genes—on the expression of a *EGFP* transgene inserted into the unique gene loading site of the alphoid^tetO^-HAC vector. Our results indicate that proximity to centrochromatin does not protect genes loaded into the HAC vector from silencing if they are not flanked by insulator sequences. Our study also demonstrates that a well-known cHS4 insulator as well as two newly discovered insulators, tDNA and gamma-satellite DNA, provide strong protective effects on transgene expression in the HAC. In two analyzed host cell lines, the highest barrier activity was observed for a tDNA insulator consisting of two functional copies of *tRNA* genes. HAC-based vectors carrying a therapeutic gene protected by any of these insulator sequences should be useful in the development of optimal strategies for stable transgenesis in different types of cells.

## Materials and methods

### Cell culture

HeLa cells were cultured in Dulbecco’s modified Eagle’s medium (DMEM; Invitrogen, Carlsbad, CA, USA) supplemented with 10 % fetal bovine serum (FBS; Atlanta Biologicals, Lawrenceville, GA, USA), 1 % penicillin–streptomycin (P/S; Invitrogen, USA) with 2 μg/ml of Blasticidin S (BS; Invitrogen) at 37 °C in 5 % CO_2_. Hypoxanthine phosphoribosyltransferase (HPRT)-deficient Chinese hamster ovary (CHO) cells (JCRB0218) were maintained in Ham’s F-12 nutrient mixture (Invitrogen) plus 10 % FBS, 1 % P/S and 8 μg/ml of BS. After introduction of the *EGFP*-containing vectors, CHO cells retaining the alphoid^tetO^-HAC-EGFP were cultured with 1× HAT medium (Invitrogen).

### Construction of the targeting vectors

The targeting vectors used in this study were constructed using standard ligation methods. Vectors #1 (CAG-EGFP), #3 (CAG-EGFP-Gamma), and #4 CAG-EGFP-tDNA were built in this study. Vector #2 (X3.1-I-EGFP-I) containing the green fluorescent protein (*EGFP*) transgene flanked on either sides by the *cHS4* insulator was built previously [20].

The vectors containing *EGFP* flanked by either tDNA or gamma-satellite DNA (gamma-satellite DNA from the human chromosome 8) [35] were constructed as follows. Two fragments of 500 bp each containing two copies of the *tRNA* gene were amplified from plasmid Rev-pCR4-TOPO [34] using a pair of primers SacI-tDNA/SpeISphI-tDNA (fragment A) and BstEIIBamH1-tDNA/NotI-tDNA (fragment B) (Table S1) and then cut by *Sac*I/*Spe*I and *Bst*EII/*Not*I, correspondingly. Vector X3.1-I-EGFP-I was digested with *Sac*I/*Spe*I and ligated with fragment A and then digested with *Bst*EII/*Not*I and ligated with fragment B, producing the final vector CAG-EGFP-tDNA (#4). The gamma-satellite DNA was isolated as two fragments of 1.9 kb each by digestion of the vector gamma8-TOPO2.1 [35] by either *Bam*H1/*Not*I or *Sac*I/*Sph*I. The *Bam*H1/*Not*I fragment was ligated with CAG-EGFP-tDNA vector digested by *Bam*HI/*Not*I. The resulting vector was then digested by *Sac*I/*Sph*I and ligated to the gamma-satellite DNA *Sac*I/*Sph*I fragment, producing the final vector CAG-EGFP-Gamma (#3).

The control vector CAG-EGFP (#1) containing the *EGFP* marker not flanked by insulators was constructed as follows. The basic vector X3.1-I-EGFP-I was digested by *Eco*RI/*Not*I and the fragment containing the 3′HPRT-loxP module was isolated from the gel (fragment A). Next, a *Eco*RI/*Not*I linker (Table S1) was ligated with fragment A, producing the intermediate vector 261ENot. A fragment of 1,768 bp containing the *CAG* promoter was excised from the X3.1-I-EGFP-I vector by *Kpn*I/*Pac*I digestion and ligated with the 261ENot vector digested by *Kpn*I/*Pac*I, producing the intermediate vector ENotCAG. A fragment of 1,273 bp containing the *EGFP* marker and the bovine globuline pA signal was PCR-amplified from the X3.1-I-EGFP-I vector using specific primers (Table S1) and then ligated with the ENotCAG vector digested by *Pac*I/*Not*I, producing the final vector CAG-EGFP. This vector contains a 3′HPRT-loxP module and the *EGFP* marker under the *CAG* promoter. In vectors #2, #3 and #4, both 5′ and 3′ insulators that flank the *CAG*-*EGFP* gene were verified by sequencing using primers that anneal outside of the insulators.

### FISH analysis of CHO cells with the BAC probe

FISH analysis was performed as previously described [20, 21]. HAC-containing CHO cells were cultured in F12 medium with 10 μg/ml of colcemid (Invitrogen) overnight at 37 °C. Metaphase cells were trypsinized and centrifugated for 4 min at 172 × *g*, treated in 10 ml of 50 mM KCl hypotonic solution for 20 min at 37 °C and washed three times in methanol:acetic acid (3:1) solution with a 4-min centrifugation at 172 × *g* between each wash. Cells were diluted to the appropriate density with fixative solution, spread onto pre-cleaned slides (Thermo Fisher Scientific, Waltham, MA, USA) above steam (boiling water), and allowed to age 2 days at room temperature. For BAC probing, CHO metaphase slides were then washed in 70 % formamide in 2× SSC for 2 min at 72 °C. Samples were dehydrated through a 70, 90, and 100 % ethanol series for 4 min each and left to air-dry. Orange 552 dUTP (5-TAMRA-dUTP) (Abbott Molecular, Des Plaines, IL, USA) labeled probes were denatured in hybridization solution at 78 °C for 10 min and left at 37 °C for 30 min. The hybridization mix probe was applied to the sample and incubated at 37 °C overnight. Slides were washed with 0.4× SSC, 0.3 % Tween 20 for 2 min at 72 °C, briefly rinsed with 2× SSC, 0.1 % Tween 20 (10 s) and air-dried in darkness. The samples were counterstained with VECTASHIELD mounting medium containing DAPI (Vector Laboratories, Burlingame, CA, USA). Slides were analyzed by fluorescence microscopy. Images were captured using a DeltaVision microscopy imaging system in the CRC, LRBGE Fluorescence Imaging Facility (NIH) and analyzed using ImageJ software (NIH). The probe used for FISH was BAC32-2-mer(tetO) DNA containing 40 kb of alphoid-tetO array cloned into a BAC vector as described previously [19]. BAC DNA was labeled using a nick-translation kit with Orange 552 dUTP (5-TAMRA-dUTP) (Abbott Molecular).

### FISH analysis of HeLa cells with PNA probes

Preparation of HeLa metaphase slides was identical to that of CHO metaphase slides with the exception that HeLa metaphase cells were obtained by overnight incubation in DMEM medium with 0.05 μg/ml colcemid (Invitrogen). For PNA probing, slides were rehydrated with 1×PBS for 15 min, and fixed in 4 % formaldehyde-1× PBS for 2 min, followed by three 5-min washes in 1xPBS, then dehydrated with a 70, 90, and 100 % ethanol series for 5 min each, and left to air-dry. PNA (peptide nucleic acid) labeled probes used were telomere (CCCTAA)_3_-Cy3) (PerSeptive Biosystems, Framingham, MA, USA) and tetO-alphoid array (FITC-OO-ACCACTCCCTATCAG) (Panagene, South Korea). Ten nanomoles of each probe were mixed with hybridization buffer and applied to each slide. Slides were then incubated at 80 °C for 3 min and hybridized for 2 h at room temperature in darkness. Slides were washed twice in 70 % formamide, 10 mM Tris–HCl; pH 7.2, 0.1 % BSA, followed by three washes with 1xTBS, 0.08 % Tween-20. Slides were then dehydrated through an ethanol dehydration series, air-dried, and counterstained with VECTASHIELD mounting medium containing DAPI (Vector Labs). Images were captured using a DeltaVision microscopy imaging system in the CRC, LRBGE Fluorescence Imaging Facility (NIH) and analyzed using ImageJ software (NIH).

### Microcell-mediated chromosome transfer

Microcell-mediated chromosome transfer (MMCT) was performed as described previously [20, 22]. The alphoid^tetO^-HACs containing the 3′ HPRT/loxP/EGFP/Insulator vectors were transferred from CHO (Chinese hamster ovary) into HeLa (human cervical carcinoma) cells via MMCT. Briefly, microcells were prepared by centrifugation of 1 × 10^9^ CHO cells attached to flasks (Nalge Nunc, USA) coated with poly-l-lysine (Sigma-Aldrich, St. Louis, MO, USA) and fused to 1 × 10^6^ HeLa cells using a Neo EX HVJ Envelope Transfection Kit (Cosmo Bio, Carlsbad, CA, USA). HeLa hybrids were selected in the presence of 2 μg/ml of blasticidin (BS) and collected for expansion.

### Genomic DNA preparation and PCR analysis

Genomic DNA for PCR analysis was prepared using DNAzol (Invitrogen). Reconstitution of the *HPRT* gene after Cre/lox-mediated recombination was confirmed by primers Lox137-R and Rev #6. Cross contamination by CHO chromosomes was quantified using hamster-specific primers, Furin-F and Furin-R. Human-specific primers dog-F1 and dog-R1 were used to verify that the fused cells were human. All these primers are listed in Table S1.

### FACS analysis

Analysis of EGFP florescence on CHO and HeLa cells was performed using an LSR Fortessa flow cytometer (BD Biosciences, Franklin Lakes, NJ, USA), with the data processed by BD FACSDiva software (BD Biosciences). A minimum of 1 × 10^4^ cells was analyzed for each sample. The intensity of EGFP florescence was calibrated using a Rainbow Calibration Particle (6.0−6.4 μm) standard (Spherotech, Inc., Lake Forest, IL, USA).

### ChIP assay and real-time PCR

ChIP with antibodies against trimethyl H3 Lys4 (Millipore, Bedford, MA, USA) was carried out using a chromatin immunoprecipitation (ChIP) assay kit (Millipore) according to the manufacturer’s instructions. Each sample used 1.2 × 10^6^ cultured cells, which were cross-linked in 1 % formaldehyde (Electron Microscopy Sciences-EMS, Hatfield, PA, USA) for 10 min at 37 °C. Soluble chromatin was prepared by two 5-min sonications at 8 °C (Bioruptor sonicator; Cosmo Bio) in lysis buffer with added protease inhibitors, yielding an average DNA size of 500 bp. We used 2 μl per sample of H3K4me3, with 2.5 μl of mouse IgG per sample used as a control for unspecific binding. The recovery ratio of the immunoprecipitated DNA relative to input DNA was measured by real-time PCR using a 7900HT Fast Real-Time PCR system (Applied Biosystems, Carlsbad, CA, USA) and Syber Green PCR master mix (Applied Biosystems). The results within each experiment were normalized to unspecific binding of mouse IgG antibody, while comparisons between different insulators was made by normalizing to rDNA, which was assumed to be unaffected by the presence of insulators in the HAC. Primers for *EGFP*, *bsr*, *Hygro*, and *HSV*-*TK* transgene sequences as well as for control loci, *5S* ribosomal DNA and pericentromeric satellite 2 repeats are listed in Supplementary Table S1. At least three independent ChIP experiments were performed to estimate the level of enrichment.

### Statistical analysis

Statistical analysis was made using Prism (GraphPad Software Inc., La Jolla, CA, USA). The data were normalized using a logarithmic transformation and a two-way ANOVA was conducted to examine the effect of the insulator type and culturing time on the intensity of EGFP florescence of the cell. A post hoc Tukey test was performed if a significant effect was found and *p* < 0.05 was considered significant.

### TSA and SAHA treatment

Clones without insulators (N1, N5, and N7 for HeLa cells and n4 for CHO cells) that have been cultured for 12 weeks were grown on a six-well plate. Once 90 % confluence was reached, the cells were incubated in 2 ml of nonselective medium containing either 100 ng/ml of Trichostatin A (TSA) (Wako) or 10 mM of suberoylanilide hydroxamic acid (SAHA) (Vorinostat) for 24 h. These cultures were then collected and analyzed by FACS.

## Results

The insulators we have tested here include the most known and widely used one, cHS4, discovered by Felsenfeld and collaborators [32]. cHS4 is derived from DNAse hypersensitive site 4 of the locus control region of the chicken β-globin gene cluster. cHS4 exhibits both enhancer-blocking and barrier activity, making it a good candidate for use in gene transfer applications. An important characteristic of the cHS4 insulator is that it functions in a vast number of vertebrate cell types and organisms tested, including rodent and human cells [29]. Other insulators have been less commonly used for experiments on gene expression in mammalian cells. Among them are two recently identified chromatin insulators, tDNA and gamma-satellite DNA, which are proposed to be essential elements in the establishment and maintenance of chromatin domain architecture in mammalian cells [33–35]. It was demonstrated that a cluster of *tRNA* genes can protect a randomly integrated transgene from silencing in human and mouse cells [33, 34]. Similarly, short tandem arrays of gamma-satellite repeats present in pericentromeric regions of all human chromosomes exhibit a strong anti-silencing activity [35]. Previously we exploited a reporter gene assay for barrier element function in mouse MEL cells [36] to demonstrate that both *tRNA* genes and gamma-satellite DNA can protect local chromatin structure by preventing heterochromatin spreading from flanking regions [34, 35].

### Construction of vectors with the *EGFP* transgene flanked by cHS4, tDNA, or gamma-satellite DNA insulators

Four vectors were used for comparative analysis of insulator activity (Fig. 1b). All vectors contain the green fluorescent protein (*EGFP*) transgene and a 3′ HPRT-loxP module designed for site-specific targeting into the single loxP gene loading site of the alphoid^tetO^-HAC using Cre/lox mediated recombinase. Vector #2 (X3.1-I-EGFP-I), in which the transgene is flanked by the full-length 1.2 kb cHS4 element on both sides, was constructed previously [29]. Two other newly constructed vectors, CAG-EGFP-Gamma (#3) and CAG-EGFP-tDNA (#4), contain the *EGFP* transgene flanked by either a 0.5-kb segment containing two *tRNA* genes (*Gly* and *Glu*) [34] or a 1.9-kb segment containing nine copies of 220-bp gamma-satellite repeat units from chromosome 8 [35]. The vector CAG-EGFP (#1) in which the *EGFP* transgene is not flanked by insulators was also constructed and used as a control. (For a detailed description of vector construction see "Materials and methods").

### Insertion of the vectors into a single loxP site of the alphoid^tetO^-HAC in hamster CHO cells

**Fig. 2:**
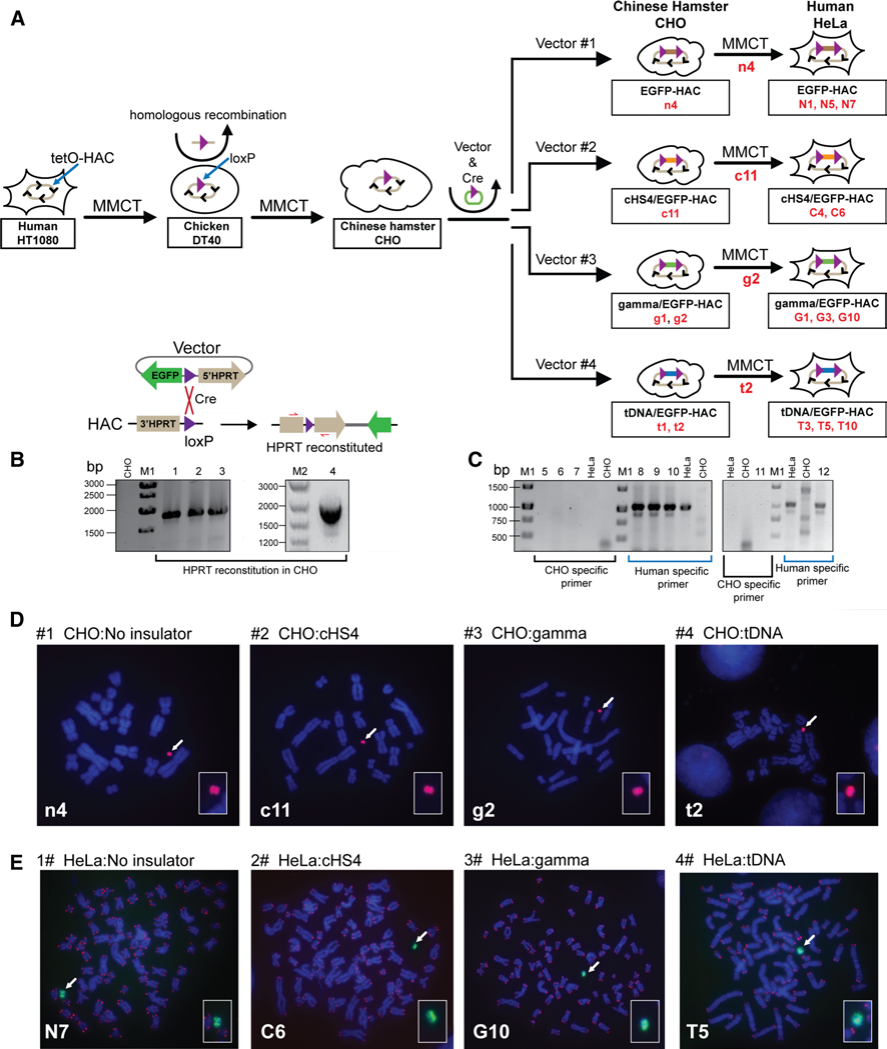
Schematic diagram of the generation of isogenic hamster and human cell lines containing the alphoid^tetO^-HACs carrying four different constructs. **a** Steps of MMCT to transfer a HAC into hamster CHO and then to human HeLa cells. The alphoid^tetO^-HAC was engineered in human HT1080 cells [19]. The loxP gene loading site was inserted into the HAC by homologous recombination in chicken DT40 cells [20]. This modified HAC was transferred to hamster CHO cells. In those cells, four vectors containing the *EGFP* transgene with or without flanking insulator elements and a 3′ HPRT-loxP module were inserted into the loxP site of the HAC by Cre-loxP mediated recombination, producing isogenic hamster cell lines. From CHO cells, autonomously propagated HACs carrying four different vectors were transferred to HeLa cells, producing isogenic human cell lines. **b** Loading of the vectors into the loxP site of the HAC propagated in HPRT-deficient hamster CHO cells is accompanied by reconstitution of the *HPRT* gene allowing cell selection on HAT medium. *Lanes 1*, *2*, *3*, and *4* correspond to PCR products obtained with the genomic DNA isolated from HAC-containing clones (n4, c11, g2 and t2) of CHO cells that were chosen for MMCT transfer to human HeLa cells. **c** PCR control for cross contamination of human HeLa cells by hamster chromosomes. *Lanes* *5*–*10* correspond to randomly chosen clones of HeLa cells containing the HAC (G10, T5, C6); clones were checked by PCR using furin-F/furin-R hamster specific primers (*lanes 5*–*7*) or by PCR using dog-F1/dog-R1 human specific primers (*lanes 8*–*10*). *Lanes* *11*, *12* correspond to the HAC-containing clone of HeLa cells (N5) checked by PCR using either hamster (*lane 11*) or human (*lane 12*) specific primers. FISH analysis of **d** the hamster clones (n4, c11, g2, t2) carrying the autonomously propagated HACs and **e** human clones (N5, C6, G10, T5) carrying the autonomously propagated HACs. FISH analysis in human cells was performed using PNA labeled probes for telomeric (*red*) and tetO-alphoid sequences (*green*). FISH analysis in hamster cells was performed using the BAC probe specific for the HAC-vector sequence (*red*) (see details in "Materials and methods"). Chromosomal DNA was counterstained with DAPI (*blue*). The HACs are indicated by *arrows*

The alphoid^tetO^-HAC containing a single loxP site [20] propagated in hamster HPRT-deficient CHO cells was used in these experiments. Each vector and a Cre-recombinase expression plasmid were co-transfected into CHO cells carrying the alphoid^tetO^-HAC (Fig. 2a). Integration of the vector into the loxP loading site within the HAC reconstitutes the *HPRT* gene, allowing cell selection on HAT medium. The recombinant clones were isolated by growth in HAT medium for 2–3 weeks. PCR analysis (Table S1) showed that the *HPRT* gene was indeed reconstituted in all drug-resistant clones (Fig. 2b). The efficiency of vector targeting into the HAC was ~1–2 × 10^−4^. All transfectants expressed the *EGFP* transgene. One EGFP-HAC clone (n4), one cHS4/EGFP-HAC clone (c11), two tDNA/EGFP-HAC clones (t1, t2), and two gamma/GFP-HAC clones (g1, g2) were identified by FISH analysis of CHO metaphase spreads and confirmed to carry autonomous HACs (Fig. 2d). These clones then were used for further analyses.

### MMCT transfer of four different alphoid^tetO^-HACs to human HeLa cells

To examine the insulator activity of cHS4, gamma-satellite DNA and tDNA in human cells, HACs carrying the *EGFP* transgene flanked by different insulators (c1, g2, t2) and without insulator sequences (n4), were transferred from hamster CHO to human HeLa cells via microcell-mediated chromosome transfer (MMCT) (Fig. 2a). HeLa cells were chosen for these experiments because the level of heterochromatin-specific proteins in these cells is higher compared to other cell lines, which leads to fast silencing of transgenes [37]. As the alphoid^tetO^-HAC contains the blasticidin selectable marker (*bsr*) [19], selection of clones was carried out with BS. Between 3 and 7 drug-resistant clones that expressed the *EGFP* transgene were isolated for each HAC. PCR analyses with a set of hamster-specific primers (Table S1) showed that there was no cross contamination by the hamster chromosomes after MMCT (Fig. 2c). FISH analysis showed that the HACs were maintained autonomously without any detectable integration into the host genome in three out of five for EGFP-HAC (N1, N5, N7), in two out of seven clones for cHS4/EGFP-HAC vector (C4, C6), in three out of three clones for gamma/GFP-HAC vector (G1, G3, G10) and in three out of five randomly selected clones for tDNA/EGFP-HAC vector (T3, T5, T10) (Fig. 2e). These clones were used for further analyses.

### Comparative analysis of EGFP expression flanked by different insulator sequences

The *EGFP* transgene expression from the HACs could be monitored by fluorescence microscopy. The level of *EGFP* transgene expression from each HAC construct was quantitated using fluorescence activated cell sorting (FACS) analysis by measuring the geometric mean green fluorescence of the cell population. Two cell lines, hamster CHO and human HeLa, carrying different HACs, were used for analysis. Each measurement was the average of values of three independent experiments for each clone. EGFP expression was measured every 2 weeks for 3–5 months.

**Fig. 3:**
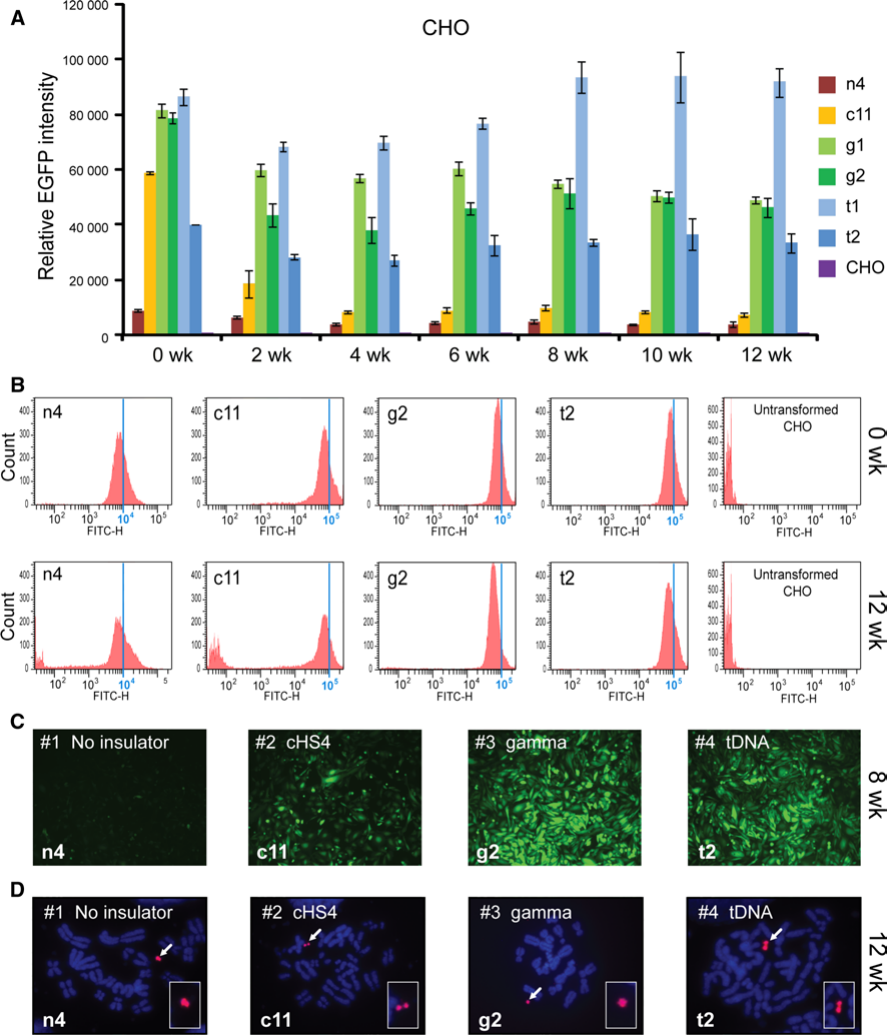
Long-term *EGFP* transgene expression in hamster CHO cells. **a** Relative mean EGFP fluorescence determined by FACS of cells carrying different HAC constructs grown under BS selection (clones n4, c11, g1, g2, t1, t2). *Error bars* represent standard deviation. EGFP-positive cells were sorted and analyzed by FACS over the course of 3 months, with measurements taken every 2 weeks. The measurement for each clone is the average of three experiments. **b** Flow cytometry histograms of levels of EGFP fluorescence in the population at the beginning of the experiment (0 weeks) and after 12 weeks of culture under BS selection. The* x*-axis represents the intensity of the fluorescence, the* y*-axis the number of cells. **c** Fluorescence images of CHO cells with the alphoid^tetO^-HAC carrying the different EGFP constructs after 8 weeks (n4, c11, g2, t2) of culture. Cells were cultured in BS^+^ medium to select for retention of the HAC. **d** FISH analysis of representative CHO clones (n4, c11, g2, t2) carrying the autonomously propagated HACs after 12 weeks of culture under selection in BS^+^ medium. Chromosomal DNA was counterstained with DAPI (*blue*). The HACs are indicated by *arrows*

For CHO cells, one clone with the EGFP-HAC construct (n4), one clone with the cHS4/EGFP-HAC construct (c11), two clones with the gamma/GFP-HAC construct (g1, g2), and two clones with the tDNA/EGFP-HAC construct (t1, t2), were analyzed by FACS. A two-way ANOVA was conducted in order to examine the effect of the insulator type and culturing time on the EGFP intensity. At the start of the experiment (0 week), all insulator constructs exhibited significantly higher EGFP expression than the control (n4) (Fig. 3a) [two-way ANOVA, F(3, 98) = 369.7; 0 week cHS4, *p* < 0.0001; 0 week gamma, *p* < 0.0001; 0 week tDNA, *p* < 0.0001]. There were also no initial significant differences between insulator constructs.

During 3 months of culturing, the intensity of EGFP expression did not change significantly and remained stable in cell lines with the transgene flanked by either gamma-satellite DNA or tDNA sequences (clones t1, t2, g1, g2) [two-way ANOVA, F(6, 98) = 15.92; gamma 0 vs. 12 week, *p* = 0.0846; tDNA 0 vs. 12 week, *p* = 0.9998]. In contrast, for the HAC construct carrying the transgene flanked by the cHS4 insulator sequence (clone c11), the geometric mean intensity of EGFP expression dropped precipitously after 2 weeks of culture (*p* = 0.0002) and remained at a low level, only twofold higher than the control (clone n4) from week 4 until the end of the experiment (week 12). This twofold difference was significant (12 week, cHS4 vs. n4, *p* = 0.0262). This drop in intensity was caused by the emergence of a population of non-florescent cells that nevertheless continued to carry the cHS4/EGFP-HAC.

In the absence of flanking insulators (control n4), the EGFP intensity decreased gradually and significantly by 2.5-fold over the course of the 12-week experiment (Fig. 3a). This decrease in EGFP intensity became significant after 10 weeks of culturing (n4, 0 vs. 10 week, *p* = 0.0131). The gradual emergence of non-florescence cells during the experiment was the cause of this decrease in the average EGFP intensity in the cell population of the clone c11. Figure 3b shows representative flow cytometry histograms of the level of EGFP fluorescence in cultures at the beginning of the experiment (0 weeks) and after 12 weeks of culture under selection for BS. Fluorescence images of CHO cells with the different alphoid^tetO^-HAC vectors (n4, c11, g2, t2) are shown in Fig. 3c after 8 weeks of culture. FISH analysis demonstrated that the HACs remained in an autonomous form in all clones analyzed after 12 weeks of culture under the HAC selection (Fig. 3d).

**Fig. 4:**
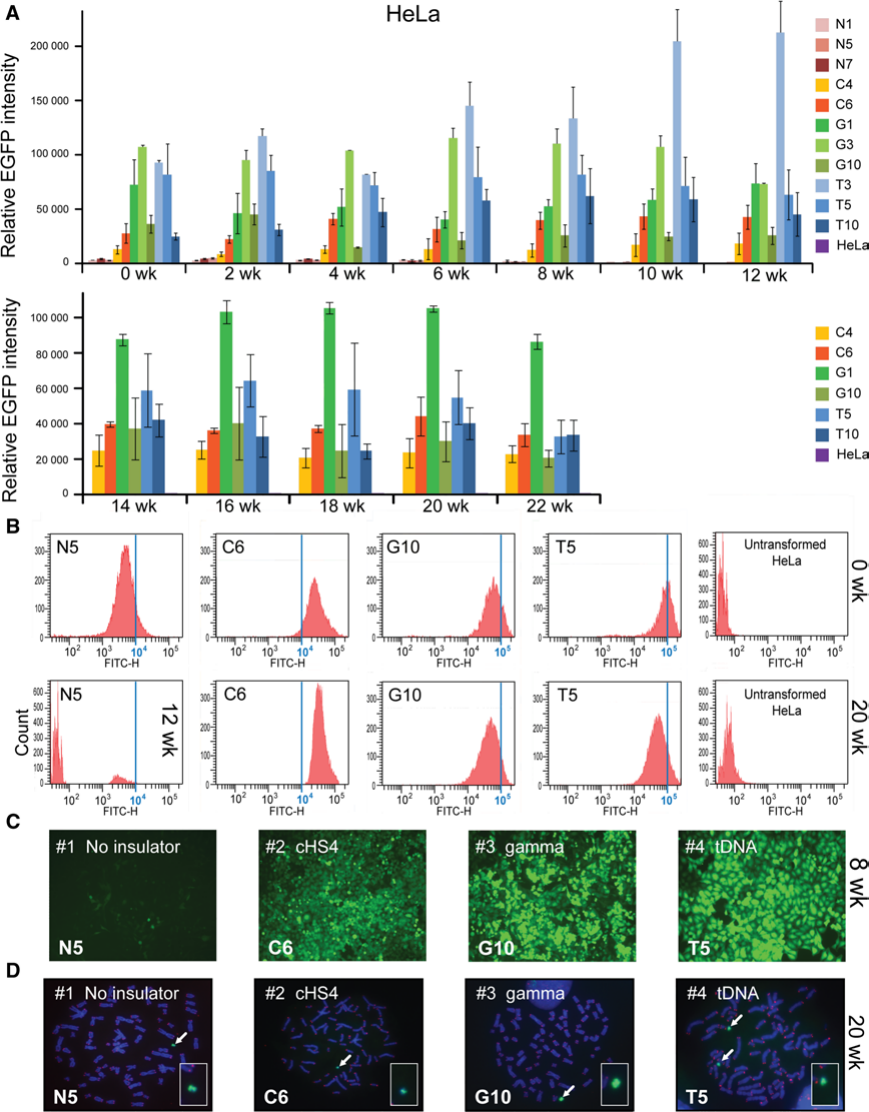
Long-term *EGFP* transgene expression in human HeLa cells. **a** Relative mean EGFP fluorescence determined by FACS of cells carrying different HAC constructs grown under BS selection similar to that described in Fig. 3 for hamster CHO cells. Each data point represents the mean ± SD from three independent experiments. Three clones for the HAC construct without insulator (N1, N5, N7), two clones for the HAC construct with chicken insulator cHS4 (C4, C6), three clones for the HAC construct with gamma-satellite DNA (G1, G3, G10) and three clones for the HAC construct with tDNA (T3, T5, T10) were cultured during 12 weeks. Two clones for each HAC construct (C4, C6, G1, G10, T5, T10) were grown for 22 weeks. Cells were cultured with HAC selection (in BS^+^ medium). **b** Flow cytometry histograms of the levels of EGFP fluorescence in the population at the beginning of the experiment (0 weeks) and after 20 weeks of culture. The* x*-axis represents the intensity of the fluorescence, the* y*-axis the number of cells. **c** Fluorescence images of HeLa cells with the alphoid^tetO^-HAC carrying the different EGFP transgenes after 8 weeks of culture (N5, C6, G10, T5 clones). **d** FISH analysis of representative HeLa clones (N5, C6, G10, T5) carrying the autonomously propagated HACs after 20 weeks of culture under selection in BS^+^ medium. Chromosomal DNA was counterstained with DAPI (*blue*). The HACs are indicated by *arrows*

For HeLa cells, three clones for EGFP-HAC (N1, N5, N7), two clones for cHS4/EGFP-HAC construct (C4, C6), three clones for gamma/GFP-HAC construct (G1, G3. G10), and three clones for tDNA/EGFP-HAC construct (T3, T5, T10), were used for analysis. As seen from Fig. 4a, at the start of the experiment (0 week) all constructs, including those with the cHS4 insulator sequence, exhibited significantly higher EGFP expression compared to the control [two-way ANOVA; F(3, 313) = 495.5; 0 week cHS4, *p* < 0.0001; 0 week gamma, *p* < 0.0001; 0 week tDNA, *p* < 0.0001]. The expression level of all insulator constructs did not change significantly after 12 weeks of culture under BS selection [two-way ANOVA; F (6, 313) = 1.808, *p* = 0.0969]. Neither did we observe any significant change in the EGFP florescence in clones C4, C6, G1, G10, T5, and T10 that were cultured longer, up to 22 weeks under BS selection [two-way ANOVA, F(11, 179) = 2.261, *p* = 0.1303]. Figure 4b shows representative flow cytometry histograms of the level of EGFP fluorescence in cultures at the beginning of the experiment (0 weeks) and after 20 weeks of culture. Unlike in CHO, no significant population of EGFP(-) cells emerged in HeLa clones with any of the three insulator constructs. Fluorescent images of one representative clone for each construct, i.e., tDNA/EGFP-HAC (T5), gamma/GFP-HAC (G10), cHS4/EGFP-HAC (C6), and EGFP-HAC (N5), are shown in Fig. 4c. FISH analysis showed that the HACs are maintained autonomously in the host genome in all clones analyzed after culturing for 20 weeks with the HAC selection (in BS^+^ medium) (Fig. 4d).

**Fig. 5:**
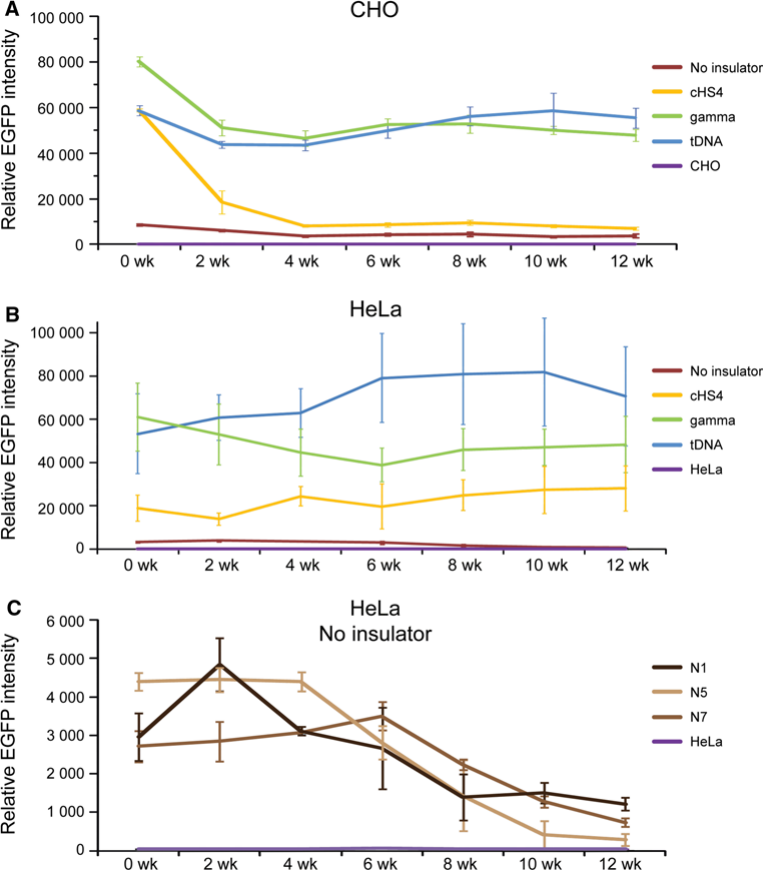
Temporal analysis of the geomentric mean EGFP intensity of clones of CHO (**a**) and HeLa (**b**) cells with the alphoid^tetO^-HAC carrying different *EGFP* transgenes (*brown* no insulator; *yellow* with cHS4; *green* with gamma-satellite DNA; *blue* with tDNA; *purple* untransformed cells) during 12 weeks of culture with selection in BS^+^ medium. **c** EGFP expression undergoes a progressive decline over the course of 12 weeks if the gene is not flanked by insulator sequences in HeLa cells. The *error bars* represent the pooled standard deviation of all clones from each insulator type

The average (geometric mean) EGFP intensity over the duration of the 12-week experiment in CHO cells was approximately 3-, 11-, and 11-fold higher for the HAC constructs carrying either cHS4, gamma-satellite DNA or tDNA sequences, respectively, compared to the control construct without *EGFP* flanking sequences (Fig. 5a). This difference in expression between the control and insulators was significant [two-way ANOVA, F (3, 98) = 369.7; cHS4, *p* < 0.0001; gamma, *p* < 0.0001; tDNA, *p* < 0.0001]. There was no significant difference in the EGFP intensity between HAC constructs carrying gamma-satellite DNA or tDNA (*p* = 0.9659). However, both gamma-satellite DNA (*p* < 0.0001) and tDNA (*p* < 0.0001)-containing HAC constructs were significantly more intense in their EGFP florescence than cHS4/EGFP-HAC, despite no initial differences at the start of the experiment. The difference in EGFP intensity between insulators in CHO emerged after 2 weeks of culture (cHS4 vs. gamma, *p* = 0.0002; cHS4 vs. tDNA, *p* = 0.017).

In HeLa cells, the average (geometric mean) EGFP intensity over 12 weeks of culture was approximately 11-, 24-, and 35-fold higher for the HAC constructs carrying either cHS4, gamma-satellite DNA or tDNA sequences respectively compared to the control constructs without EGFP flanking sequences (Fig. 5b) [two-way ANOVA, F (3, 313) = 495.5; cHS4, *p* < 0.0001; Gamma, *p* < 0.0001; tDNA, *p* < 0.0001]. In HeLa cells, the HAC constructs carrying tDNA had significantly higher EGFP florescence than gamma-satellite DNA constructs (*p* = 0.0001). Both gamma-satellite DNA (*p* < 0.0001) and tDNA (*p* < 0.0001) -containing HAC constructs were also significantly brighter than cHS4/EGFP-HAC. In the control cell lines without insulators (N1, N5, N7), a growing population of EGFP(-) cells began to emerge at 6 weeks of culturing. This caused a significant decrease in the average EGFP intensity of the cell population by 12 weeks [two-way ANOVA; F (6, 313) = 1.808, *p* < 0.0001] (Fig. 5c).

To summarize, the level of the EGFP expression during a 3-month period in hamster (CHO) and a 5-month period in human cells (HeLa) was highest when the *EGFP* transgene was flanked by either gamma-satellite DNA or tDNA sequences. In both cell types, the presence of insulators significantly increased the intensity of the EGFP florescence.

**Fig. 6:**
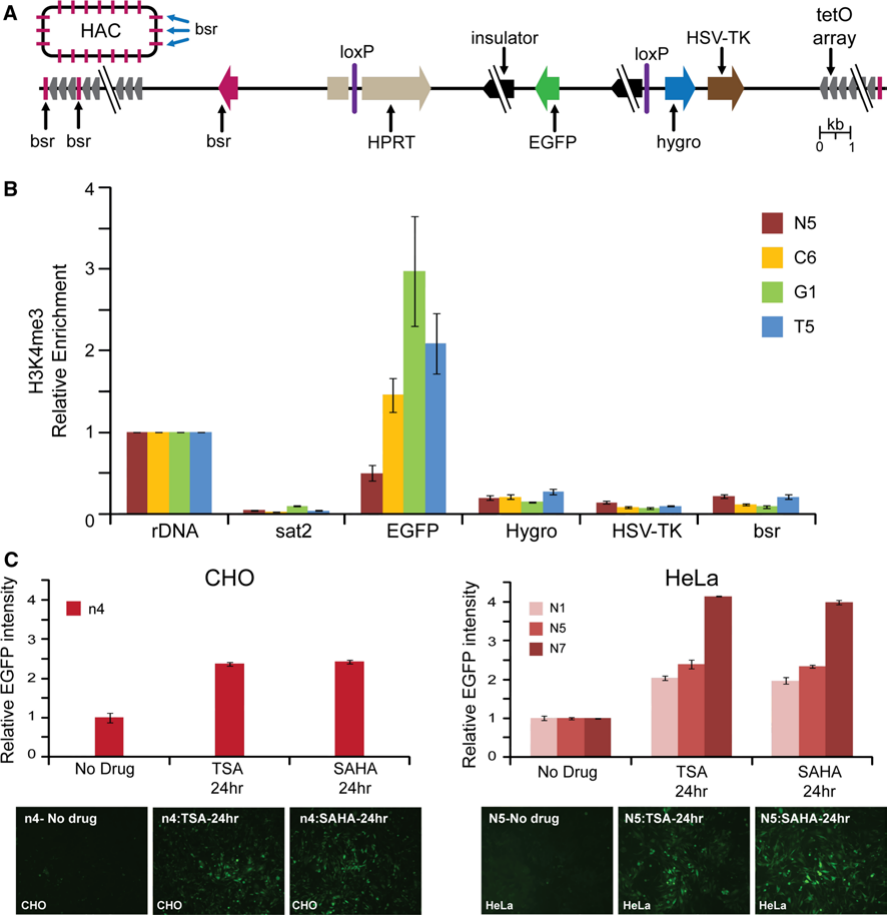
ChIP analysis of H3K4me3 chromatin in the transgene cassettes on the alphoid^tetO^ HAC propagated in HeLa cells. **a** A map of the transgenes within the HAC; HSV-*TK*, *Hygro*, *HPRT* genes* plus* an *EGFP* color marker flanked on both sides by different insulators. The *bsr* gene is repeated 30–40 times within the 1.1 Mb tetO-alphoid array of the alphoid^tetO^-HAC [19, 21]. *Arrows* indicate the direction of transcription of the transgenes. **b** ChIP analysis was performed for clones C6, G1, T5, and N5. Enrichment is shown relative to the *5S rRNA* control locus. Satellite 2 sequence, *Sat2,* from endogenous pericentromeric repeats was included as a negative control. **c** The n4 clone of CHO and N1, N5, and N7 clones of HeLa cells were treated either with TSA or SAHA. EGFP intensity is shown relative to the non-treated cultures. Fluorescence images of cells with and without drug treatment are shown. In the CHO control cell line (n4), a significant increase in GFP intensity was observed upon treatment with either TSA or SAHA [one-way ANOVA, F (2, 15) = 589.0; TSA, *p* < 0.0001; SAHA, *p* < 0.0001]. There was no significant difference in GFP intensity between TSA- and SAHA-treated cells (*p* = 0.4523). A similar pattern was observed in HeLa control cell lines N1, N5, and N7. GFP expression was significantly increased in HeLa cells upon drug treatment. [one-way ANOVA, F (8, 27) = 1287; N1-TSA, *p* < 0.0001; N1-SAHA,* p* < 0.0001; N5-TSA, *p* < 0.0001; N5-SAHA, *p* < 0.0001; N7-TSA, *p* < 0.0001; N7-SAHA, *p* < 0.0001]. No significant difference was detected between TSA- and SAHA-treated cells (N1, *p* = 0.4615; N5, *p* = 0.9597; N7, *p* = 0.0502)

Chromatin immunoprecipitation (ChIP) analysis was employed to analyze the chromatin structure of the alphoid^tetO^-HAC DNA carrying different cassettes in human HeLa cells. For this experiment, we examined three HAC clones (C6, G1, T5) after 19 weeks of culture and one HAC clone (N5) after 6 weeks of culture. More specifically, the HAC region containing the single copy *EGFP* transgene, *Hygro*, and *HSV*-*TK* genes as well as the *bsr* gene (which was repeated ~30–40 times within the mega-size alphoid DNA array) [19] were analyzed (Fig. 6a). ChIP analysis revealed that H3K4me3, a marker for active gene promoters, was highly associated only with the *EGFP* transgene sequence flanked by cHS4 or tDNA or gamma-satellite DNA sequences (Fig. 6b). A very low association was observed for the *bsr* gene sequences scattered throughout the megabase-size alphoid array of the HAC as well as for *Hygro* and *HSV*-*TK* genes not protected by insulator sequences. We assume that the lower levels on the *bsr* genes reflect the fact that most copies of this gene are likely to be inactive on the HAC. These results clearly indicate that cHS4, tDNA, as well as gamma-satellite DNA sequences work as barrier elements protecting a transgene against heterochromatin domains on the alphoid^tetO^ DNA array of the HAC. A statistical difference in enrichment of H3K3me3 on EGFP between insulators was determined by one-way ANOVA. A Tukey post hoc test revealed that enrichment of H3K4me3 on EGFP in the C6 clone (cHS4) was statically significantly lower than in either G1 (gamma-satellite DNA) [F(3, 12) = 50.81, *p* < 0.0001] or in T5 (tDNA) (*p* = 0.0274) clones. There were no statistically significant differences between gamma-satellite DNA and tDNA (*p* = 0.557). All three insulator constructs were significantly higher than the control (N7) (C6, *p* = 0.0002; G1, *p* < 0.0001; T5, *p* < 0.0001). This pattern is similar to that of the FACS results.

*EGFP* gene silencing in control clones N1, N5, and N7 in HeLa cells and in the control clone n4 in hamster CHO cells is likely to be caused by heterochromatinization of the *EGFP* region initiated from nearby alphoid DNA sequences of the HAC. Indirect support for this conclusion comes from experiments where these clones containing the *EGFP* transgene without insulator protection were treated with the histone deacetylase (HDAC) inhibitors, TSA (trichostatin), and SAHA (suberoylanilide hydroxamic acid) [38]. EGFP expression was reactivated when these inhibitors were added to the culture (Fig. 6c, d), suggesting that an increase in acetylation level of histone H3 in the HAC favors transcription of the transgene.

## Discussion

The expression of transferred exogenous genes is subject to the influence of surrounding chromatin. The phenomenon of “chromosomal position effects” frequently leads to rapid and irreversible transgene silencing. Several studies have shown that flanking a transgene with chromatin insulators can reduce both the rate and extent of transgene silencing and position effect variegation [29–31].

The cHS4 insulator derived from the chicken β-globin locus has been intensively used to protect transgene expression [32]. Barrier activity of the native cHS4 element is associated with a peak of histone hyperacetylation and other hallmarks of an “open” histone modification profile. These are thought to prevent the invasion of transcriptionally repressive heterochromatin from an adjoining region into the chicken β-globin locus [39]. Subsequent studies demonstrated that this activity is associated in part with the binding of a protein complex, including USF1 and other histone modifying enzymes, at specific points within and adjacent to the cHS4 core region [40].

A similar mechanism, but involving other protein complexes, has been proposed for two other insulators identified in human genomes, tDNA and gamma-satellite DNA. The barrier activity of tDNA requires binding of Pol III factors and their interaction with chromatin remodeling and histone modifying enzymes in a spational organization of these loci in a nuclear zone [33, 34]. Interestingly, *tRNA* genes have long been known to flank the central core of centromeric DNA in *S. pombe* [41].

In contrast to *tRNA* genes scattered in the human genome, gamma-satellite DNA is localized exclusively in the pericentromeric regions of all human chromosomes. Gamma-satellite DNA arrays at their natural location may act to prevent heterochromatin spreading beyond the pericentromeric regions. Anti-silencing activity of gamma-satellite insulator depends on the binding of Ikaros [35], which promotes chromatin remodeling and activation of target genes in pericentromeric chromatin [42].

Based on accumulating literature data, a level of gene “protection” by an insulator depends both on the nature of the insulator sequence and on the chromatin organization of the regions neighboring the insulator [30, 31]. Therefore, the effect of insulators on expression of genes loaded into de novo formed HAC vectors cannot be absolutely predicted. In the case of the alphoid^tetO^-HAC, our previous chromatin analyses indicated that our synthetic gene-loading site is inserted into a transcriptionally permissive centrochromatin domain [22, 28]. In the HACs as well as in centromeres of normal chromosomes, this domain is flanked by heterochromatin domains essential for centromere function [43]. Based on such organization, two scenarios could be considered. First, centromeric chromatin may represent a privileged region for Pol II-transcribed genes, as was previously shown for rice centromeres [44]. In this case, insulators may not be required at all for stable gene expression in this region. Alternatively, genes located in centrochromatin may undergo silencing—caused either by specific features of centrochromatin that support only a very low level of transcription on the underlying centromeric repeat sequences [23, 26] or by repositioning of heterochromatin domain(s) after replication of centromeric DNA.

In this study, using a set of isogenic cell lines carrying the alphoid^tetO^-HAC vector, we investigated the effect of three chromatin insulators, cHS4, and gamma-satellite DNA and tDNA, on the expression of the *EGFP* transgene. Our results indicate that epigenetic silencing of the unprotected transgene occurs when it is in close proximity to centrochromatin. Formally, this is in agreement with both (i) the hypothesis of spreading of heterochromatin domain(s) within a functional centromere, and (ii) with a repressive effect of centrochromatin domain. We prefer the first alternative based on the kinetics of extinction of expression of the insulator-less transgene. A low level of EGFP expression observed just after gene loading progressively declined during subsequent cell culturing (Fig. 5c), as might be expected if there is a gradual heterochromatization of the transgene sequence (but not if centrochromatin represses transgene expression from the outset).

With all three insulators, transgene expression remained at a relatively high level, with no evidence of significant silencing in human cells. The highest barrier activity was detected for a tDNA insulator consisting of two functional copies of *tRNA* genes and gamma-satellite DNA repeats. It is worth noting that a similar effect was observed when these chromatin insulators were analyzed at an ectopic chromosomal site in mouse cells [34, 35]. Thus, chromatin insulators positioned in centrochromatin function similarly to their action previously reported at other chromosomal locations.

The strong protective effect of insulators observed in this study indicates that chromatin insulators should be included in future experiments on long-term gene expression in HACs. These chromatin insulators could be inserted into the HAC along with a gene of interest. For example, insulators can be included in the retrofitting vector pJBRV1 [22] that has been specifically constructed for loading full-length genes isolated by transformation-associated recombination (TAR) cloning [1–3] into the alphoid^tetO^-HAC. Because the activity of an insulator may vary in different cells, it would be practically useful to flank a gene of interest with different insulators and test which is more suitable for a specific host cell environment. Notably, the HAC-based vector described here may also be used to discover new barrier elements that prevent gene silencing in centrochromatin.

### Electronic supplementary material

Below is the link to the electronic supplementary material.

Supplementary material 1 (DOC 28 kb)
