# Supplementary material for: Protecting a transgene expression from the HAC-based vector by different chromatin insulators
Source: Cell Mol Life Sci. 2013 May 16;70(19):3723–37. doi: 10.1007/s00018-013-1362-9 (PMC3771377; doi:10.1007/s00018-013-1362-9)
Supplement: Supplementary file 1 — Supplementary material 1 (DOC 28 kb) [file 18_2013_1362_MOESM1_ESM.doc]

**Table S1 Primers used in this study**

Oligonucleotide name Sequence (5’-3’) Endonucleases

***Construction of vectors***

SacI-tDNA-F 5′ CGGAGCTCGCGCCAATCCCATTGCAAATTCTAC 3′ SacI

SpeISphI-tDNA-R 5′ GGACTAGTGCATGCTGCATTCCACACACAAGGTG 3′ SpeI

*Eco*RI/*Not*I linker 5’ gcggccgcacgctccagacccatcattaattaattctcgacggtatcgataagcttgataattcttaggtaccgaattc 3’

***Primers for HPRT reconstitution***

Lox137-R 5′ AGCCTTCTGTACACATTTCTTCTC 3′

Rev #6 5′ GCTCTACTAAGCAGATGGCCACAGAACTAG 3′

***Primers for CHO DNA contamination after MMCT***

Furin-F 5’ ACTCAGAGATCCACTGCACCAGGATCCAAGGGAGG 3’

Furin-R 5’ CCGCTCGAGCGGCTACACCACAGACACCATTGTTGGCTACTGCTGCC 3’

***Human specific primers for SPANX-L gene after MMCT***

dog-F1 5’ TGTGGAATCTCATTGTCCTGAG 3’

dog-R1 5’ GTTCTCAACTCCCTCCCTCCT 3’

***Primers for qPCR***

***BSD gene***

bsr-F 5′ TCCATTCGAAACTGCACTACCA 3′

bsr-R 5′ CAGGAGAAATCATTTCGGCAGTAC 3’

***5S Ribosomal DNA***

rDNA-F 5′ ACGCTGGGTTCCCTGCCGTT 3′

rDNA-R 5′ TGGCTGGCGTCTGTGGCACCCGCT 3′

***Satellite 2***

Sat2-F 5′ TCGCATAGAATCGAATGGAA 3′

Sat2-R 5′ GCATTCGAGTCCGTGGA 3′

***EGFP***

GFP-F 5′ AGAACGGCATCAAGGTGAAC 3′

GFP-R 5′ AGCTCAGGTAGTGGTTGTCG 3′

***Hygro***

Hygro-f2 5′ TTGTTGGAGCCGAAATCCG 3′

Hygro-rev2 5′ CAAACTGTGATGGACGACACCG 3′

***TK***

TK-f2 5′ GCGTCGGTCACGGCATAAG 3′

TK-rev 2 5′ GGGTGAGATATCGGCCGGG 3′
